# In the eye of the stakeholder: The challenges of governing social forest values

**DOI:** 10.1007/s13280-015-0745-6

**Published:** 2016-01-07

**Authors:** Anna Sténs, Therese Bjärstig, Eva-Maria Nordström, Camilla Sandström, Clas Fries, Johanna Johansson

**Affiliations:** Department of Historical, Philosophical and Religious Studies, Umeå University, 901 87 Umeå, Sweden; Department of Political Science, Umeå University, 901 87 Umeå, Sweden; Department of Forest Resource Management, SLU, Skogsmarksgränd, 901 83 Umeå, Sweden; Swedish Forest Agency, P.O. Box 284, 901 06 Umeå, Sweden

**Keywords:** Cultural ecosystem services, Forest management, Legal instruments, Multiple use forestry, Social values, Stakeholder analysis

## Abstract

This study examines which kinds of social benefits derived from forests are emphasised by Swedish stakeholders and what governance modes and management tools they accept. Our study shows that there exists a great variety among stakeholders’ perceptions of forests’ social values, where tourism and recreation is the most common reference. There are also differences in preferred governance modes and management where biomass and bioenergy sectors advocate business as usual (i.e. framework regulations and voluntarism) and other stakeholders demand rigid tools (i.e. coercion and targeting) and improved landscape planning. This divide will have implications for future policy orientations and require deliberative policy processes and improved dialogue among stakeholders and authorities. We suggest that there is a potential for these improvements, since actors from almost all stakeholder groups support local influence on governance and management, acknowledged and maintained either by the authorities, i.e. targeting, or by the stakeholders themselves, i.e. voluntarism.

**Electronic supplementary material:**

The online version of this article (doi:10.1007/s13280-015-0745-6) contains supplementary material, which is available to authorised users.

## Introduction

…I initially thought foresters managed physical things. It requires deeper and more detached thinking to look through these things and see the social values behind them. (Kennedy 1985)

Despite a long-standing ambition to introduce the concepts of social and cultural values into international and national forest policy, these aspects of sustainable forest management (SFM) remain the least developed. For different reasons, such measures are perceived as very challenging to govern (e.g. Agnoletti et al. 2008; Boström 2012). This article will discuss the status of social forest values and different approaches to their current and future governance in a Swedish forest management context.

The concept of multiple use management was introduced in North American and German forest policy during the 1950s and 60s, when resources such as recreation, water, wildlife and fisheries were officially re-introduced as important aspects to consider in addition to timber production. Similar elements were included in forest policy in most Nordic countries in the 1970s (Hytönen 1995). In Sweden, for example, the Forestry Act of 1979 stated that: “Forestry must be conducted with regard to the importance of forests to plants and animals, water balance and local climate as well as for outdoor activities and recreation. Valuable cultural heritage sites and the visual quality of the landscape must be considered.” (SKSFS 1979). Hence, forests’ social and cultural values were enhanced and driven to reflect recreation, cultural heritage and landscape aesthetics.

Exploitation of forests for industrial purposes was however still prioritised in many countries, including Sweden. Due to continued criticism against the industrial use of forests, SFM approaches were introduced during the 1980s and early 90s, not least as a response to the release of the Brundtland report *Our Common Future* in 1987 and the United Nations Conference on Environment and Development (UNCED) meeting in Rio de Janeiro 1992 (e.g. Johansson 2013). Following demands to foster sustainable development, many states including the Nordic countries adjusted their forest policies to further enhance ecological and social values by giving them equal priority to economic values (Hytönen 1995; Kankaanpää and Carter 2004). The Forest Principles approved by the UNCED in 1992, which still provide foundations for the idea of SFM world wide, state that forest resources should be managed to meet the social, economic, ecological, cultural and spiritual needs of present and future generations (UNCED 1992). More recently, the concept of ecosystem services has helped further raise attention to forests’ cultural and social benefits (Abson et al. 2014). Cultural ecosystem services are defined as “the nonmaterial benefits that people obtain from ecosystems through spiritual enrichment, cognitive development, reflection, recreation, and aesthetic experiences” (MA 2003, p. 58).

Despite the recognition of these values, SFM policy has so far focused on the preservation and enhancement of ecological functions of forests for economic productivity. Politicians, stakeholders and researchers have found forests’ social and cultural values particularly hard to analyse, comprehend and define, and there is little agreement on how to include them in the current system of measurable goals, criteria and indicators that permeates environmental policy (Agnoletti et al. 2008; Boström 2012; Chan et al. 2012). Social and cultural considerations in forest management are however crucial to sustainable development. Management that considers the different aspects of cultural heritage, traditional knowledge and recreation, can help to improve diversification and competitiveness of marginal rural economies. It enhances both ecological conditions and the appearance of landscapes and can eventually help communities to achieve a higher quality of life (Agnoletti et al. 2008).

In this article, we focus on descriptions of current and future governance of forests’ social values in Sweden by assessing opinions of organised stakeholders who take part in forest policy processes. Organised interests play an important role in forest policy processes and their views will have implications for future policy and management orientations. As in many other European countries, most (ca. 80 %) of Swedish forests are privately owned (Swedish Statistical Yearbook of Forestry 2014), and strong private property rights are combined with generous public access rights, the so-called “allemansrätten”, to all forests regardless of ownership. Hence, generally wood production coexists with recreation and various other activities on the same land (Sténs and Sandström 2013). Furthermore, a core element of both Swedish and Finnish forest policy is a high degree of flexibility, encapsulated in the notion of ‘freedom with responsibility’, which presupposes a willingness of owners and users to take various kinds of voluntary action to meet objectives of SFM (Sandström et al. 2011). A particularly important example of voluntary arrangements for Swedish forest management is certification. The growth of certification was spurred by the failure to adopt an international forest convention at the UNCED in 1992, which subsequently induced non-state actors to initiate private alternatives in order to halt unsustainable forestry practices. Approximately 50 % of the total productive forest area in Sweden is certified by the Forest Stewardship Council (FSC) and the Programme for the Endorsement of Forest Certification (PEFC), under schemes that include certain criteria relevant to forests’ social values (Johansson 2013).

Recently, public interest in forests’ social values has increased in Sweden, partially due to a formal acknowledgement of the importance of outdoor recreation, followed by authorities’ recognition of a lack of knowledge regarding the conditions for outdoor recreation in Swedish forests (e.g. Bladh et al. 2014). A series of articles (Zaremba 2012) criticising forestry for still catering too little for “ordinary” people’s interest in and feelings for forests also fuelled the debate (e.g. Stridsman 2012; Larsson 2012). Authorities have tried to elucidate what currently signifies forests’ social values and how they should be protected and/or developed. The latest official policy on forests’ social values departs from the framework of cultural ecosystem services and describes them as mainly non-material values created by people’s “experiences” of forests in dimensions such as health, recreation, knowledge, social relations, inspiration, identity and cultural heritage (Birkne et al. 2013). A closer look at these new policy formulations however suggests that forests’ social values are more or less exclusively understood as recreation and tourism. The same pattern is found on the Nordic level where efforts have been made to enhance recreational values within the European SFM policy framework (Sievänen et al. 2013).

On the other hand, national certification standards represent a broader understanding of social values, rooted in the international principles for SFM mentioned above. This understanding encompasses both non-material and material objectives, such as the desire to “secure people’s livelihoods, promote a safe environment for workers, respect the cultures of local populations and Sami people” and consider wildlife, fungi, berries, fish and recreation (FSC 2010; cf. PEFC 2012, under revision in 2015). The distribution of social values and their impacts in Sweden have however received rather little empirical attention in the certification literature (Johansson 2013). Previous research on social aspects of certification has nonetheless found that stakeholders representing civil society and local communities merely play a consultative role in decision-making and have limited access to monitoring and evaluation (Roberge et al. 2011). Others observe a lack of real decision-making power for indigenous peoples despite their strong formal position in the FSC standard (Sandström and Widmark 2007).

Hence, there is no consensus on how to describe forests’ social values on a national policy level. While the ecosystem services framework drives public authorities, the certification schemes lean on international principles for SFM. This potentially confuses the discussion on forests social values among actors involved in the policy debate. However, our first hypothesis is that there is an even wider spectra of values associated with Swedish forests among stakeholders that goes well beyond these currently established definitions in forest policy. Our second hypothesis is that there are equally conflicting views on how to govern forests, including their social values, among Swedish stakeholders and that these reflect the common divide between forestry and other interests.

The aim of this article is thus twofold: the first is to examine to what extent stakeholders who are key in national forest policy processes agree on descriptions of social values, and the second is to assess what kind of instruments of governance and management of these values they are willing to accept. We provide an empirical overview based on a review of the different stakeholders official policy documents and a complimentary survey conducted by e-mail.

The results of the study will be applicable to perceptions, policies and responses in countries where large proportions of forested land are privately owned and governed by soft law.

## Governing forests’ social values

The concept of governance has come to dominate scholarly and political debates on sustainable forests (Agrawal et al. 2008). The concept includes various forms of practices through which forests are governed and is often distinguished from the notion of governing, which can be defined as actions that make a “purposeful effort to guide, steer, control, or manage sectors or facets of societies” (Kooiman 1993, p. 2). Governing is thus related to government and the formal institutions of the state, whereas governance includes both institutional forms of governing and non-hierarchical forms of steering through any kinds of network or other arrangements across states, markets and civil societies (Kooiman 1993; Rhodes 1997; Stoke 1998; Pierre and Peters 2000). Due to the changes in governing outlined above, there is now a variety of co-existing modes of forest governance promoting or supporting different types of relationships between governmental and non-governmental actors through binding and non-binding legal instruments. However, the concept of governing covers not only the nature of state-societal arrangements, but also how policy or legislation is implemented and the types of regulatory instruments applied (Lange et al. 2013).

In the scholarly debate, there is an on-going normative discussion about the change, i.e. its desirability and how much it has actually impacted the steering capacity of the state. These state-centric or society-centric viewpoints are reflected in the debate about the forest governance system, as some stakeholders promote more top-down forms of steering while others support various non-hierarchical modes of governance such as decentralisation, public–private partnerships, co-management or privatisation. A number of studies have explored the Swedish governance system from this perspective (e.g. Schlyter and Stjernquist 2010; Sundström 2010; Appelstrand 2012). Other studies have explored how the system is influenced by evolving international institutions (Lindstad and Solberg 2012; Bjärstig 2013; Bjärstig and Keskitalo 2013), and how it is affected by the changing values, attitudes and practices of forest users (Eriksson et al. 2013). Several studies discuss how market-driven tools such as certification systems (Boström 2003; Widmark 2009; Johansson 2013) and collaborative and voluntary instruments affect the Swedish forestry model (Appelstrand 2012; Klenk et al. 2013; Widman 2015). Most of these studies however focus on ecological and economic values, while few studies incorporate the governance of social values (cf. Sténs and Sandström 2013).

In order to analyse how stakeholders conceptualise forests’ social values and the governance modes they promote, it is necessary to operationalise various modes of governance for analytical purposes. This typology of governance modes builds on Treib et al. (2007), where we illustrate the different modes with our own specific examples relating to forests.

Legal provisions are assumed to be either binding or non-binding, and implementation to be rigid or flexible. This results in four ideal types of governing: coercion, targeting, framework regulation and voluntarism (Table 1) (cf. Knill and Lenschow 2003; Treib et al. 2007; Sténs and Sandström 2013). Thus, forests’ social values may be governed coercively (via binding legal instruments with detailed rules regarding resource access and management) or, at the other extreme, through voluntary guidelines such as certification schemes. In the former case, public legislators have to make decisions on matters such as whether social and cultural values should be defined and managed in general terms, and how policies should be implemented and monitored, e.g. by punishing forest owners who destroy hiking trails or expropriating areas to preserve them for recreational use. Among the traditional policy tools of ‘sticks‘, ‘carrots’ and ‘sermons’ (Vedung et al. 1998), the focus would primarily be on sticks, i.e. regulatory instruments. In the latter case, the non-state stakeholders involved define common policy goals and how they are to be achieved, as in the international FSC and PEFC forest certification schemes. The other two ideal types—targeting and framework legislation—both focus on defining overarching goals. Framework legislation, such as the current Swedish Forestry Act (1979), builds upon binding policy goals adopted by Parliament but allows designated agents (in this context the Swedish Forest Agency) some leeway in implementation through issuing rules and recommendations. Conversely, targeting relies on policy collaboratively developed by government and stakeholders, as in the Finnish and Swedish processes of establishing National Forest Programmes or the development of forest management objectives (Andersson et al. 2013). It offers more details on how things should be done, i.e. the means of achieving objectives, through processes such as nature conservation agreements, but promotes a bottom-up perspective. Hence, voluntarism and targeting rely on more incentive-based policy instruments such as the aforementioned ‘carrots’ and ‘sermons’.

**Table 1:** A typology of governance modes (Treib et al. 2007)

	Binding	Non-binding
Legal instruments		
Rigid	Coercion: regulation by a detailed national legislation. Implementation by sticks (strong enforcement, penalties, expropriation, centralised top-down planning).	Targeting: policy goals or standards are set by the government and stakeholders in collaboration, specifying how goals are to be met. Implemented through decentralised agreements and partnerships.
Flexible	Framework regulation: National policy regulating overarching policy goals. Leeway in implementation, i.e. “Freedom with responsibility”, sermons (information) and carrots (economic incentives).	Voluntarism: policy, both in terms of setting goals and implementation, is dealt with voluntarily by the actors involved through e.g. certification schemes. Implementation relies on private initiatives.

In the forest, the different governance modes are realised through forest management and planning. In the coercive mode of governing, regulations concerning forest management and social values may be quite strict and detailed, so that some silvicultural measures are prescribed, such as natural regeneration, while others such as clear-cutting are prohibited. In this mode, forest management plans complying with a standard set by the state may be mandatory for the forest owners. Consequently, objectives set by the state may have precedence over the forest owners’ objectives for forest management. In contrast, under voluntarism, forest owners are free to set objectives in their forest planning according to their own interests or a voluntary standard like a certification scheme. They then manage their forests in the ways they believe are optimal to meet these objectives (unless a certification scheme is applied, which may include quite detailed forest management prescriptions). In the current mode of governing, by framework regulation, overarching production and environmental goals are set for forest management, but silvicultural measures are not regulated in much detail. Forest plans are not mandatory but the authorities encourage forest owners to develop such plans, which are mainly pushed towards meeting forest owners’ objectives, provided that production and environmental goals of the Forestry Act are not violated. In a targeting mode of governance, forest plans are developed collaboratively by forest owners and stakeholders, and the state may provide ways to meet policy goals.

## Materials and methods

Our study is based on a stakeholder analysis, undertaken to identify actors, or groups of actors, who have stakes in Swedish forests. The term stakeholder refers to “all those who affect, and/or are affected by the policies, decisions and actions” (Grimble and Chan 1995, p. 114) related to, in our case, forests’ social values. We chose to include organised, non-governmental stakeholders, who represent interests that are involved as consultant or referral bodies in national forest policy processes, for example, in the forest authorities attempt to formulate appropriate guidelines for social values (e.g. Birkne et al. 2013). This resulted in a list of 25 stakeholders (Supplementary Material S1). All stakeholders, except the Swedish Church and the Swedish Landowners’ Association, also participated in the scenario analysis component of the Future Forests programme (Sandström et al. 2016). Dissimilar to the scenario analysis, stakeholders in this study are sorted in seven categories, reflecting principal interests instead of frames, including Biomass & Bioenergy, Conservation, Hunting & Fishing, Tourism & Recreation, Sami Livelihood, Cultural Heritage and Rural Development. We found these narrower categories useful for assessing and displaying the differences among actors and interests in the forest policy process.

Sources of analysed data include published policy documents, policy-related information on websites and published consultative opinions on issues relevant to forests’ social values. Some of these documents do not explicitly speak about social values but indirectly. Subsequently, an e-mail survey was sent to the stakeholders where they were asked to define what they perceive as social values of forests and what policy instruments (i.e. governance modes) they would accept to enhance these values in the future, all to assure as valid information as possible for the study (Supplementary Material S1). Since we were interested to show which benefits are the most frequently associated with forests’ social values and who makes these associations, we chose to translate qualitative statements into quantitative data. Stakeholders’ descriptions of forests’ social values were thus extracted from the sources, and all key values and activities mentioned were tabulated. The descriptions often include a number of values and activities, e.g. tourism and recreation, berry-picking and health. All the aspects mentioned were included, and to obtain a better overview, most of the aspects were grouped into categories of social benefits from forests as recognised by researchers and policies on ecosystem services and SFM (Bryan et al. 2010; De Groot et al. 2010). Aspects that did not fit into any of these categories were left distinct (cf. Supplementary Material S2). UCINET open source software (UCINET 2015) was then used as a tool to visualise the results, showing the most to least common categories of social benefits currently associated with forests.

A similar approach was applied when analysing stakeholders’ attitudes to different governance modes. The stakeholders’ expressed preferences regarding governance modes are not always consistent. In their policy documents, for example, conflicting methods and tools are often promoted. Thus, we have selected those representing the most rigid type articulated/accepted by each stakeholder as presented in Table 2.

**Table 2:** Swedish stakeholder categories’ views on legal instruments and policy implementation

	Binding	Non-binding
Legal instruments		
Rigid	Coercion: Conservation Hunting & Fishing Tourism & Recreation Sami Livelihood	Targeting: Biomass & Bioenergy Conservation Hunting & Fishing Sami Livelihood Cultural Heritage Rural Development
Flexible	Framework regulation: Biomass & Bioenergy Hunting & Fishing Tourism & Recreation Rural Development	Voluntarism: Biomass & Bioenergy

The stakeholders’ views on forest management and planning were also analysed. Forest management was defined so as to include statements on both stand-level silvicultural and harvesting activities (e.g. planting, scarification, pre-commercial thinning, thinning, final felling, etc.) and forest management systems (e.g. even-aged forestry and continuous cover forestry). The concept of forest planning was defined as planning and implementing silvicultural activities on estate or landscape level, i.e. the process of determining and scheduling the activities to carry out in each stand.

## Results

### Current descriptions of forests’ social values

**Fig. 1:**
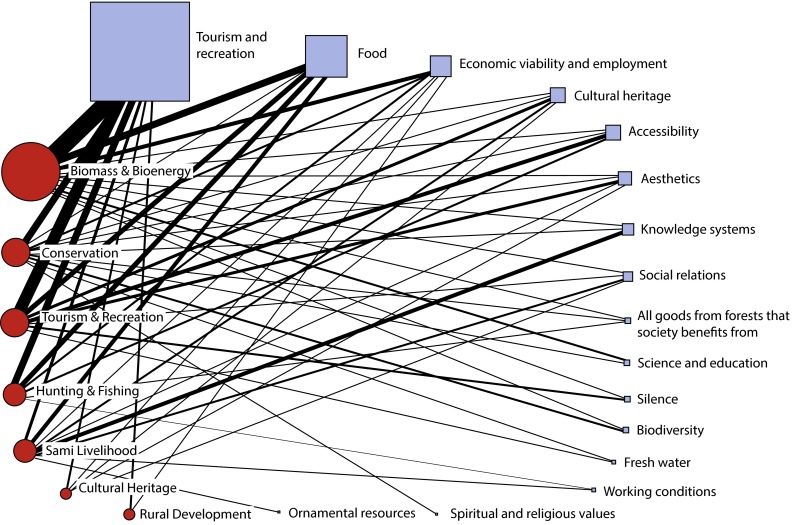
Swedish stakeholders categories’ descriptions of forests’ social values

The most common values and activities included in descriptions of forests’ social values among Swedish stakeholders are shown in Fig. 1. The red nodes represent stakeholder categories, sized according to numbers of organisations represented in each category; Biomass & Bioenergy, for example, includes nine organisations and Cultural Heritage only one. The blue nodes to the right show types of activities and values emphasised as social by the different stakeholders, sized and sorted by popularity. Thus, the figure shows that forests’ social values are most commonly connected to tourism and recreation. Recreation in particular is regarded as a social value, but it is a broad concept including *inter alia* “health” and “experiences” (for an overview of our categorisation, see Supplementary Material S2).

Products like berries, mushrooms, game and fish are also considered as social values by most stakeholders. Hence, food products from forests are not only seen as a provisioning service in accordance with the ecosystem framework, but also as values connected to tourism, recreation and economic viability. To organisations representing Sami Livelihood, the ability to extract different kinds of traditional food products, including reindeer (*Rangifer tarandus*), from forests is regarded both as an industry and essential for their knowledge system and cultural survival (e.g. the Sami Parliament).

Other popular concepts in descriptions of forests’ social values are economic viability and employment, cultural heritage and aesthetics. Economic viability and employment are strongly connected to rural development and (thus) to international policies and certification schemes addressing the importance of forests for social sustainability (e.g. Federation of Swedish Family Forest Owners; National Association of Huntsmen). Accessibility is also emphasised in several descriptions, mainly connected to the concept of right to public access, but also to availability through trails, paths and forest roads (the latter emphasised by Biomass & Bioenergy actors) and the special needs for recreational forests near urban areas.

The widest description presented is that of social values being “all goods from forests that society benefits from” (Federation of Swedish Family Forest Owners; Future Earth; National Association of Huntsmen). Several of the other less common concepts used to describe social values are connected to indigenous rights and well-being. These are mainly supported by the indigenous Sami group, with allies among Conservation actors. Working conditions are rarely mentioned by any stakeholder.

### Governance of forests’ social values

Several of the abovementioned values are already regulated by law, such as consideration of reindeer herding activity (legislatively defined not as a social non-material value but as an economic livelihood), hunting and fishing (however, not in the Forestry Act) and cultural remnants, with stronger regulations for remnants from before year 1850. *Allemansrätten* is seen as a customary right, protected by the Swedish constitution. However, tourism and recreation, aesthetics, traditional knowledge and spiritual values are vaguely regulated or neglected by legislation, though they are acknowledged in forest certification schemes. A key issue is thus how these social values should be governed according to the stakeholders.

Using the typology of governance modes presented in Table 1, we categorised the stakeholders’ views on legal instruments and policy implementation according to the four ideal types: coercion, targeting, framework regulation and voluntarism (Table 2). The results indicate that stakeholders in the Conservation, Hunting & Fishing (chiefly anglers), Tourism & Recreation, and Sami Livelihood (as reindeer herders) are critical of the prevailing governance mode and would like more coercion, perceiving a need for stronger regulations and more protected areas to enhance the social and cultural values of forests. The Swedish Society for Nature Conservation (SSNC, Conservation) believes that freedom with responsibility has failed as a policy tool to meet the forest policy objectives, and perceive severe shortcomings in the policies and regulations governing the management of social values (SSNC 2013). Accordingly, they suggest development in several areas including legislation, economic instruments, protection, counselling, alternative management methods, mapping, guidance in planning, valuation of social values, responsibility and collaboration. The WWF is also critical, advocating changes to the Forestry Act that promote more active management to enhance forests’ environmental, social and cultural values. They also see a need to introduce opportunities to obtain injunctions, and to strengthen the Swedish Forest Agency’s role and resources for law enforcement, field assessments and provision of advice. Thus, they advocate greater centralisation of governance. The anglers’ society holds the view that forestry currently has a negative impact and that freedom with responsibility does not work satisfactorily, concluding that: “There is a need for mandatory instruments” (Sport Anglers, e-mail survey, February 18, 2015).

Targeting has a particularly broad support and is mainly advocated by Sami Livelihood and other actors promoting a decentralised governance mode with strong adaptation to local conditions, such as Future Earth (Conservation), Swedish Association for Hunting and Wildlife Management (Hunting & Fishing) and Holmen Forest (Biomass & Bioenergy). Biomass & Bioenergy actors alone promote voluntarism. A common argument posed is that forestry’s voluntary efforts always exceed what current regulation requires, often through their commitment to certification schemes (cf. Federation of Swedish Family Forest Owners; Forest Industries; SCA Forest).

However, as indicated in Table 2, most stakeholder categories show inconsistency in their reasoning regarding desirable legal instruments and policy implementation. Particularly when discussing policy implementation, they describe a whole set of complementary (and sometimes conflicting) policy tools. This is exemplified by framework legislation and coercion being supported as legal instruments, but with much less support being expressed for these approaches as means of policy implementation (for which targeting has support from actors in all groups except Tourism & Recreation). The greatest inconsistency is within the Biomass & Bioenergy category, where several of the stakeholders prefer framework regulation as a legal instrument, but voluntary measures for policy implementation. We conclude that members of this group support the current governance system of freedom with responsibility, with the Forestry Act providing framework regulations and forestry companies voluntarily committing to certification schemes deliberated by non-state stakeholders, hence flexible governance tools. Scepticism towards more binding regulations is however common among other stakeholders as well. As a representative of one organisation concludes: “Freedom with responsibility provides future benefits, while legislation preserves ideas expressed by research and politics of a specific era”. (National Association of Huntsmen, e-mail survey March 16, 2015).

To summarise the situation, it can be said that representatives from all stakeholder categories express a need for rigid governance modes. There is though a broader support for non-binding, but still rigid policy implementation (targeting) than for binding regulations implemented by “sticks” (coercion). In contrast, many actors representing landowner interests advocate flexible tools and favour voluntary incentives such as certification schemes for policy implementation. They clearly state that it is important to respect the individual forest owners’ rights to use their own forests, and that economic compensation is essential if such arrangements as mandatory considerations are made (e.g. Federation of Swedish Family Forest Owners; National Association of Huntsmen). Hence, as hypothesised, there are conflicting preferences for governance modes between the Biomass & Bioenergy group and other stakeholders. On the other hand, most of the stakeholders involved, including Biomass & Bioenergy stakeholders, desire non-binding instruments for implementation, indicating the divide may not be so strong after all.

### Planning and management for social values

Most of the stakeholders prescribe methods for planning and managing forests’ social values. A common opinion is that social values would benefit from varied management (e.g. National Association of Huntsmen; Federation of Swedish Family Forest Owners; Swedish Outdoor Association). Continuous cover forestry rather than clear-cutting is advocated by actors in the Conservation, Tourism & Recreation, and Sami Livelihood (SSNC; WWF; Swedish Outdoor Life; SSR). There is broad support for a landscape perspective in forest management (e.g. SSR; Swedish Local Heritage Association; Federation of Swedish Family Forest Owners; Holmen Forest; WWF; SSNC). This has been considered important for a long time and may grow increasingly important in the future, e.g. to implement the European Landscape Convention. However, planning covering estates of multiple forest owners and requiring a collaborative approach is controversial due to strong property rights (Fries et al. 1998). Consequently, Biomass & Bioenergy actors support landscape “perspectives” of forest management, but oppose landscape “planning”, since they are not willing to subordinate private forest owners to public requirements for coordination between different landowners (e.g. Federation of Swedish Family Forest Owners). Regarding planning for recreational values, the stakeholders present different views on whether the landscape should be divided into zones or if these values should be considered in general, i.e. all over the landscape. Zoning, with great consideration to recreational values near urban areas and less in rural, has a broad support and gather actors from the Conservation group as well as the Biomass & Bioenergy group (e.g. SSNC; Swedish Forest Industries Federation; Federation of Swedish Family Forest Owners; Swedish Orienteering Federation).

Almost all statements about forest planning for social values emphasise the need for communication with and involvement of stakeholders in the planning processes, i.e. use of non-binding instruments that could encourage a landscape perspective. However, there are again varying views regarding which stakeholders should be involved and the optimal level of participation, which again reflect the preferred modes of governance among the stakeholders (i.e. targeting versus voluntarism).

## Discussion

Our study shows that different stakeholders have different understandings of what should be considered as forests’ social values. This was an expected outcome, since as Romm (1993) implies, the definition of sustainable forests in general is an issue of what–where–when–how–who. Furthermore, forests’ social and cultural values are bound to temporal and spatial contexts more strongly than ecological and economic values and reflect views, interests and experiences of individuals and diverse social, cultural, political and economic groups, institutions and organisations (cf. Agnoletti et al. 2008). Hence, what can be considered as social values, and more importantly what might be considered as essential social values, is to a large degree in the eye (and interest) of the beholder, or as this study suggests, in the eye of the stakeholder (cf. Dussauge et al. 2015).

Nonetheless, despite this constructivist conclusion, the stakeholders surveyed in this study mirror to a large degree international definitions of social and cultural values, taking a number of material and non-material aspects into account, including recreation, employment, cultural heritage, aesthetics, social relations, biodiversity, fresh water and ornamental resources. There is also a traceable path-dependency present in their views, since concepts central to past national discussions of forests’ social values are still common, such as their importance for economic viability, employment and rural development (cf. Koch and Kennedy 1991).

Thus, as hypothesised, the views of stakeholders involved in this study go far beyond the Swedish authorities’ current definition of forests’ social values, which focus mainly on peoples immaterial “experiences” of forests such as well-being and recreation. Still, tourism, recreation and food were the most common references among our surveyed stakeholders. This does not mean that all stakeholders rank these as the most important categories, but that concepts related to these categories are mentioned most frequently in the sources. The apparent prerequisite of recreation and food as the major social values should however be critically scrutinised. Both recreation and food are to a large extent connected to ideas of romantic and utilitarian aspects of outdoor life with close ties to urbanity, (male) gender, (middle) class and national identity in Sweden and other countries in the global north (e.g. Cronon 1996; Satterfield et al. 2013; Lisberg Jensen and Ouis 2014).

Internationally, tourism and recreation also turns out to be the most scientifically explored of the less tangible goods from forests. Compared to other categories of social and cultural values, there are also well-developed methods for measuring tourism and recreation and assessing them from a monetary point of view (Hernández-Morcillo et al. 2013). This makes them adequate to the current classification system of goals, criteria and indicators that permeate SFM policy, where politicians and researchers constantly look for ways of quantifying all values from forests, in order to make them commensurable (e.g. Chan et al. 2012; Hernández-Morcillo et al. 2013; Satterfield et al. 2013). Swedish authorities’ increased priority of forests’ recreational values have recently resulted in a number of institutional changes. For example, it has become possible for state and local governments to make agreements with landowners to protect forests with high recreational values, in parallel to nature conservation efforts (Swedish Forest Agency 2014).

In contrast to recreation, working conditions are almost completely ignored by our stakeholders, although they are major issues in international definitions of SFM. This also illustrates how forest values are linked to historical context. In the 1960s and 70s, working conditions would probably have been mentioned more frequently, due to concerns about high frequencies of injuries among forest workers (cf. Synvoldt 2011). In the future, this concern might raise again as working conditions are poor for increasing numbers of seasonal migrant workers in the forestry sector (e.g. Schierup et al. 2015; Wingborg et al. 2015). However, no stakeholders acknowledge the migrants’ situation in the analysed material of this study. They are also neglected in the Swedish authorities’ definition of forests’ social values (Birkne et al. 2013).

Our second hypothesis was that there would be conflicting views on how to govern forests, including their social values and that these would reflect the common divide between forestry and other interests. The study shows that this hypothesis was less correct, since stakeholders from all categories promote deliberative processes and hence more non-binding forms of governance. None of the actors however are entirely consistent in their promotion of favoured forms of governance and policy instrument and there is still a discernible divide, where mainly Conservation and Tourism & Recreational actors included in the study promote stronger top-down regulation and implementation while Biomass & Bioenergy actors alone support voluntarism (Table 2). There is also a divide where the Biomass & Bioenergy actors to a high degree embrace deliberative processes enforced by FSC and PEFC, while other stakeholders promote deliberation through more explicit collaboration between authorities and stakeholders, i.e. targeting.

The strong support of targeting among our stakeholders is interesting since this is a new combination of policy tools in a Swedish context, involving the stakeholders interpreting the legislation through deliberative practice and implementation guidelines. Targeting can thus be seen as a hybrid between government and governance (Arts and Buizer 2009), where authorities and non-state actors voluntarily establish objectives and specify how they should be implemented jointly. Deliberative processes have their pros and cons. If poorly facilitated, strong interests often dominate, or the lowest common denominator is identified as other ideas and interests get excluded from the process (Dryzek 2000). Representatives of the environmental movement appear more critical to deliberative processes, either if enforced by certification or authorities, probably because of experiences of previous deliberative processes as being exclusionary, resource demanding and lacking focus (cf. Sandström and Sténs 2015). If properly managed, this type of process has the advantage that it may involve many stakeholders, opening up possibilities to foster diverse types of values from a bottom-up perspective. Thus, it is typically a favoured tool for mapping out the diversity of social and cultural values among local stakeholders in policy processes (Agnoletti et al. 2008; Chan et al. 2012).

Attitudes to planning tools also reflect the divide between targeting and voluntarism. The planning processes and tools currently used in Swedish forest management generally depend strongly on the forest owners’ interests, and so does planning related to social values. During the 1980s and early 1990s, forest management plans were compulsory in Sweden, but since 1994, forest planning has been voluntary and commonly initiated by the forest owner. However, according to both FSC and PEFC standards, all certified forest estates larger than 20 hectares must have a forest management plan (FSC 2010; PEFC 2012). Forest management plans for non-industrial private forest owners are usually produced by the Forest Agency, a forest owner association or another consultant. The plans could potentially promote social values, but tend to be quite standardised, being mainly oriented towards timber production with ca. 5 % of the area set aside for nature conservation. The Forest Agency has developed a model for creating management plans focused on recreation and urban forests (Eriksson 2005). The extent of its use in practice is unknown, but Lundquist (2005) found that many municipalities have recreation-adapted management plans, and the proportion has probably increased since then.

Forest companies apply a hierarchical forest planning process, first creating long-term plans setting harvest levels, and subsequently tactical (medium-term) and operational (short-term) plans (Nilsson et al. 2012). Ecological landscape plans have been included in their long-term planning since the mid-1990s, and are mandatory according to the FSC standards. Social values have not been explicitly included in the long-term planning but rather considered during operational planning in the field.

Computerised tools, e.g. the forest planning system Heureka (Wikström et al. 2011), are increasingly used by forest companies and to some extent non-industrial private forest owners. These tools provide opportunities to consider trade-offs between multiple values in long-term planning and to share information on forest management plans.

Forest management plans are implemented through silvicultural measures like planting, thinning and harvesting in forest stands. According to the Forestry Act, these measures must not, however, be adjusted to any larger extent to sustain forests’ attractiveness or recreational values (SKSFS 2011). However, considerations should be made for ancient and cultural remains (Ulfhielm 2014). Although people’s ratings of forests’ attractiveness vary, some features are consistently rated highly and could thus be better considered in silvicultural operations. According to a review by Gundersen and Frivold (2008), these include large trees, inclusion of broadleaved trees in conifer-dominated stands, ease of access and walking, water and water courses, openings in the forest cover (especially those related to former human activities), and paths. Most people dislike large clear-fellings but small ones that are well-adjusted to the landscape are sometimes welcomed. There are numerous references to the importance of all these features in Swedish forestry regulations, public recommendations and the certification schemes but recommendations of silvicultural adjustments are vague. Silvicultural measures in areas where the Sami people have territorial rights are more regulated and consultations already compulsory (cf. FSC 2010; PEFC 2012; SKSFS 2011).

## Conclusions

In 1985, Kennedy described professional foresters’ shock induced by having to cope with concerns about the social and environmental values of forests. Neither society nor forestry was prepared to handle the conflicts aroused by industrial forest management. Now, decades later, we would claim that societies, including forest sectors, are much better equipped to govern and manage forests for multiple purposes.

There is a considerable interest in forests’ social values and numerous ideas about planning tools and silvicultural regimes to promote them, some of which are enshrined to various degrees in regulations. Currently we see a positive promotion of recreational values. This is very encouraging, but society should not limit its interpretation of social values to only resemble recreation, but be aware of all those other less established or neglected aspects, that may be regarded as important by different actors in society today and in the future.

Existing planning tools and practices could be implemented far more extensively and further developed, including, for example, by formulation of more creative and customised management plans that include a spectrum of locally relevant aspects. However, application of many of these tools and methods currently relies on voluntarism, and thus also on the degree to which they coincide with the forest owners’ interests.

An overall finding of this study is that many stakeholders in Swedish forests want to maintain non-binding forms of governance, but in combination with the inclusion of more rigid forms of implementation through collaboration, decentralised agreements and partnerships. Stakeholders representing Biomass & Bioenergy also pose that collaboration is important, even if they generally urge that stakeholders themselves should be in charge of such processes and that the landowners should always be in charge of what happens to their land. Nevertheless, whoever initiates a deliberative process, it still requires sufficient competence to implement it in a way that meets all the key criteria, such as openness, and transparency of motives (for an overview, see Zachrisson 2009). It also requires the ability to use the full spectrum of policy as well as management tools and methodologies to meet the full range of social values held by diverse stakeholders. Ensuring that such competence and ability is present is far from straightforward, and in its absence, there are high risks that the most powerful interests will dominate and follow their own narrow interests. It can thus be said that there is a challenging yet promising future for the governance of forests’ social values.

## Electronic supplementary material

Supplementary material 1 (PDF 610 kb)
