# Supplementary material for: In the eye of the stakeholder: The challenges of governing social forest values
Source: Ambio. 2016 Jan 7;45(Suppl 2):87–99. doi: 10.1007/s13280-015-0745-6 (PMC4705066; doi:10.1007/s13280-015-0745-6)
Supplement: Supplementary file 1 — Supplementary material 1 (PDF 610 kb) [file 13280_2015_745_MOESM1_ESM.pdf]

Ambio

Electronic supplementary material

This supplementary material has not been peer reviewed

Title: **In the eye of the stakeholder: The challenges of governing social forest values**

Authors: Anna Sténs, Therese Bjärstig, Eva-Maria Nordström, Camilla Sandström, Clas Fries, Johanna Johansson

Supplementary material 1 Categories, actors and sources included in the study.
* Actors included who do declare a view on forest governance, but not on social values.
** Actor included who declare a view on social values, but not on forest governance.

**Categories and actors**	**Sources**
1. Biomass & Bioenergy	
Bergvik Skog* [Bergvik Forest]	Consultative opinion: Remiss fördjupad utvärdering av Levande skogar [Comments on the official report regarding the deeper evaluation of Living Forests] (2015).
Holmen Skog [Holmen Forest]	Riktlinjer för uthålligt skogsbruk [Guidelines for sustainable forestry] (2011).
LRF Skogsägarna [The Federation of Swedish Family Forest Owners]	Nära till skogen [Close to the forest] (2014) + e-mail survey, February 18, 2015.
Skogsindustrierna [Swedish Forest Industries Federation]	Levande skogar [Living forests] (2014).
SCA Skog [SCA Forest]	Skogens sociala värden [The social values of forests] (2015) + e-mail survey February 24, 2015.
Spillkråkan [The Black Woodpecker]	E-mail survey February 20, 2015.
Svebio* [The Swedish Bioenergy Association]	Consultative opinion: Remissyttrande över fördjupad utvärdering av Levande skogar [Comments on the official report regarding evaluation of Living Forests] (2015) + e-mail survey February 18, 2015.
Svenska Kyrkan [Swedish Church]	Miljö- och skogsskötselpolicy för skogsmark inom prästlönetillgångarna Svenska kyrkan [Environmental- and silvicultural policy for forestland owned by the Swedish church] (2015).
Sveriges Jordägareförbund* [The Swedish Landowners' Association]	Sveriges Jordägareförbunds grundsyn [The Swedish landowners' association's basic view], http://www.jordagarna.se/?page=grundsyn&pid=212 (Accessed May 17, 2015).
2. Conservation	
Framtidsjorden [Future Earth]	E-mail survey March 6, 2015.
Fältbiologerna** [Nature and Youth	Nyfiken på skog? [Curious about forests?]

Sweden]	(2005).
Swedish Society for Nature Conservation (SSNC)	Säkerställ skogens sociala värden [Secure forests' social values] (2013); Människan i skogen: det skogspolitiken glömde [People in the forest: what forest policy forgot] (2013).
World Wildlife Fund (WWF)	Livfulla skogslandskap och ekosystembaserat nyttjande: WWFs vision för svenska skogen 2050 [Lively forest landscapes and ecosystem-based use: WWF's vision for the Swedish forest 2050] (2013).
3. Hunting & Fishing	
Jägarnas Riksförbund [The National Association of Huntsmen]	E-mail survey March 16, 2015.
Svenska Jägareförbundet [The Swedish Association for Hunting and Wildlife Management]	75 år i allmänhetens tjänst för det vilda [75 years in public service for the wild] (2013); Consultative opinion: Kunskapsplattform för skogsproduktion [Knowledge platform for forest production] (2014); Skogsstyrelsens förstudie om ett nationellt skogsprogram för Sverige [Comments on the official report regarding the Swedish Agency's pilot study about a National Forest Program for Sweden] (2014); Kunskapsplattform för hållbart brukande av skog [Knowledge platform for sustainable use of forest] (2011).
Sportfiskarna [The Sport Anglers]	Website http://www.sportfiskarna.se/ (Accessed 2015- 04-20) + e-mail survey February 18, 2015.
4. Tourism & Recreation	
Friluftsfrämjandet [Swedish Outdoor Association]	Friluftsfrämjandets Friluftsmiljöprogram [Swedish Outdoor Association's outdoor environmental program] (2006).
Svenska Orienteringsförbundet [The Swedish Orienteering Federation]	Orienteering and the right of public access (2015) + e-mail survey March 16, 2015.
Svenska Turistföreningen [The Swedish Tourist Association]	Riktlinjer för Svenska Turistföreningen i turism-, frilufts-, natur- och kulturfrågor [Guidelines for the Swedish Tourist Association regarding tourism-, outdoor life-, nature- and culture-issues] (2015) + e-mail survey May 8, 2015.

Svenskt Friluftsliv [Swedish Outdoor Life]	Friluftslivet och samhället [Outdoor life and soicety] (2008); Consultative opinion: Remissvar översyn av föreskrifter och allmänna råd för 30§ SVL - del 2 [Comments regarding the official report Overhaul of prescriptions and common advice in 30§ Swedish Forestry Act, part 2] (2013); Remissvar: Långsiktigt hållbar markanvändning del 1 (SOU 2013:43) [Comments regarding the offical report on Long-term sustainable land use, part 1] (2013).
5. Sami Livelihood	
The Sami Parliament	Samiskt kulturlandskap [Sami cultural landscapes], http://www.sametinget.se/30605; Rennäring och skogsbruk, [Reindeer industry and forestry] http://www.sametinget.se/1129; Renen och skogen [The reindeer and the forest] http://www.sametinget.se/30605 (Accessed 2015-04-08).
Same Ätnam	E-mail survey February 25, 2015.
Samiid Riikaseravi [National Association of Swedish Sami, SSR]	Ett renskötselanpassat skogsbruk [A forestry adjusted to reindeer management] (Accessed September 11).
6. Cultural Heritage	
Sveriges Hembygdsförbund [The Swedish Local Heritage Federation]	Policy för det svenska skogslandskapet [Policy for the Swedish forest landscape] (2013).
7. Rural Development	
Hela Sverige ska leva [The Swedish Village Action Movement]	Consultative opinion: Långsiktigt hållbar markanvändning del 1, yttrande, [Comments regarding the offical report on Long-term sustainable land use, part 1] October 4, 2013 + e-mail survey February 18, 2015.

Supplementary material 2 Categorisation of social values deployed in the study.

Values	*Aspects included*	*References for categorisation*
Accessability	right of public access, forest roads	FSC Sweden 2015, Criteria 2.2; PEFC Sweden 2015, Standard 3.
Aesthetics	residential environment, living conditions, visual qualities	De Groot et al. 2010; Bryan et al. 2010.
All goods from forests that society benefits from		Left distinct.
Biodiversity	biodiversity	FSC Sweden 2015, Principle 6; PEFC Sweden 2015, Standard 2 and 4.
Cultural heritage and identity	cultural heritage, cultural values	De Groot et al. 2010; Bryan et al. 2010.
Economic viability and employment	rural development, economic maintenance, employment	Bryan et al. 2010.
Food	wildlife, hunting, angling, berries, mushrooms, herbs, grouse, reindeer	De Groot et al. 2010; Bryan et al. 2010.
Fresh water	fresh water	Bryan et al. 2010.
Knowledge systems	indigenous rights, traditional knowledge, reindeer husbandry, way of life, human right	Bryan et al. 2010.
Ornamental resources	handicraft materials	De Groot et al. 2010; Bryan et al. 2010.
Science and education	outdoor education, play, bird watching	De Groot et al. 2010.
Silence	silence	Left distinct.
Social relations	strengthen public and community involvement, spirit of community	Bryan et al. 2010.
Spiritual and religious values	indigenous rights	Bryan et al. 2010.
Tourism and recreation	recreation, tourism, exercise, walking, skiing, hunting, angling, leisure activities, snowmobiling, attractions, sports, experiences, health, well-being, recovery, happiness	De Groot et al. 2010; Bryan et al. 2010.
Working conditions	working conditions	FSC Sweden Principle 4; PEFC Sweden 2015, Standard 3.

REFERENCES

Bryan, B.A., C. M. Raymond, N. D. Crossman, and D. Hatton Macdonald. 2010. Targeting the management of ecosystem services based on social values: Where, what, and how? *Landscape and Urban Planning* 97: 111–122.

De Groot, R. S., R. Alkemade, L. Braat, L. Hein, and L. Willemen. 2010. Challenges in ntegrating the concept of ecosystem services and values in landscape planning, management and decision making. *Ecological Complexity 7*: 260–272.

FSC. 2010. Swedish FSC standard for forest certification including SLIMF indicators. Forest Stewardship Council Sweden, Uppsala. Retrieved 7 June, 2015, from https://se.fsc.org/preview.svensk-skogsbrukstandard-fsc.a-771.pdf (in Swedish).

PEFC 2012. Svensk PEFC Skogsstandard [Swedish PEFC Forest Standard]. 2012–2017. PEFC SWE 002:3. Svenska PEFC. Retrieved November 26, 2015, from http://pefc.se/wp-content/uploads/2010/11/n-pefc%20swe%20001%20-%20svenskt%20pefc%20certifieringssystem%20fr%20uthlligt%20skogsbruk%20120801.pdf (in Swedish).
